# Supplementary material for: Exploring the Effects of Rearing Densities on Epigenetic Modifications in the Zebrafish Gonads
Source: Int J Mol Sci. 2023 Nov 6;24(21):16002. doi: 10.3390/ijms242116002 (PMC10647740; doi:10.3390/ijms242116002)
Supplement: Supplementary file 1 [file ijms-24-16002-s001.zip › ijms-2646860-revised-supplemental figure.pdf]

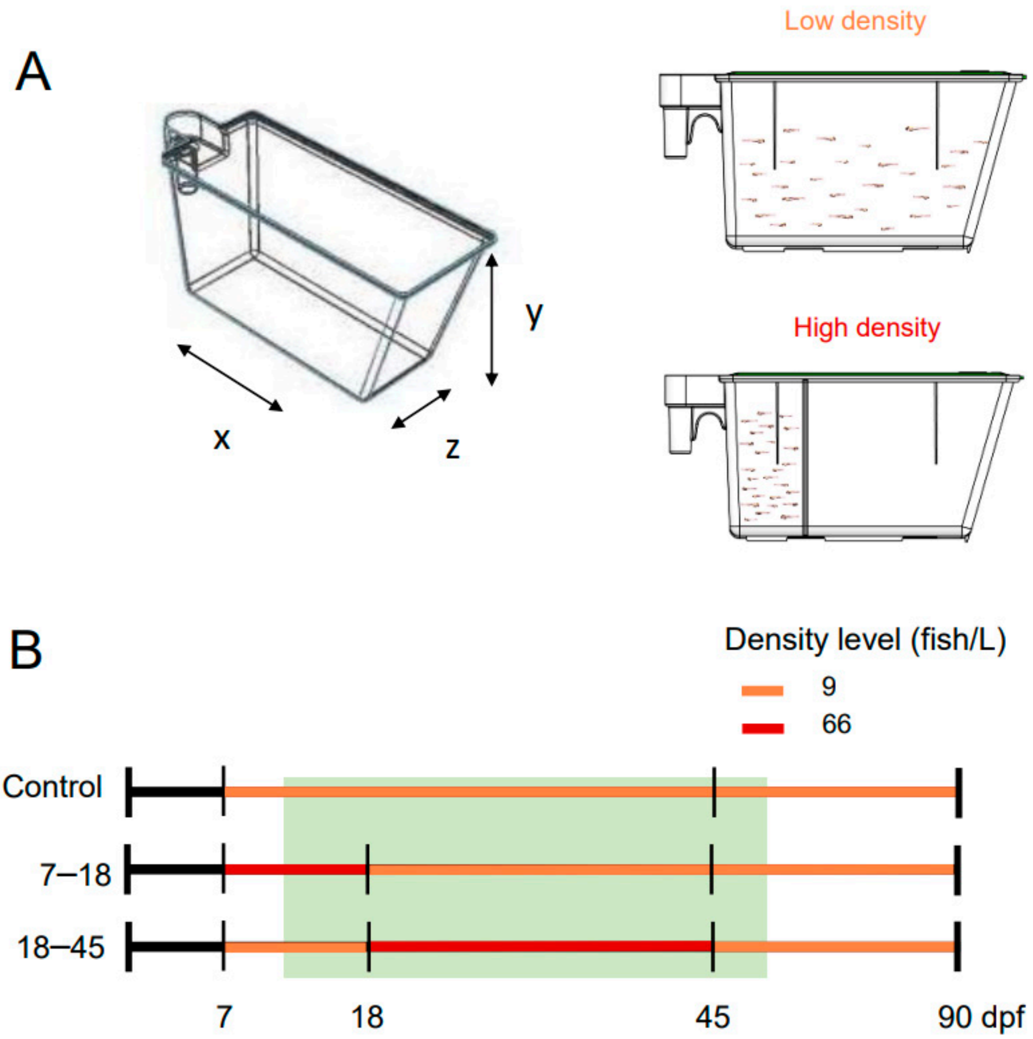

Figure S1. Experimental design to induce stress by density. **A)** Tanks used for this experiment (2.8 L volume, ZT280, Aquaneering) and how the animals were confined to control or high-density conditions. **B)** Experimental design which consisted of control, 7–18 days post fertilization (dpf), and 18–45 dpf, in which for the control group, tanks started with 9 fish/L at 7 dpf and the density level remained unmodified until 90 dpf; in 7–18 group, tanks started with 66 fish/L at 7 dpf and fish remained confined until 18 dpf; in 18–45 group, larvae were set up at 9 fish/L until 18 dpf, and then larvae were confined at 66 fish/L until 45 dpf and set up at 9 fish/L until 90 dpf. A shaded green area means the sex differentiation during gonadal development.
